# *Rickettsia sibirica mongolitimonae* Infection, Turkey, 2016

**DOI:** 10.3201/eid2307.170188

**Published:** 2017-07

**Authors:** Ferit Kuscu, Omer Orkun, Aslihan Ulu, Behice Kurtaran, Suheyla Komur, A. Seza Inal, Damla Erdogan, Yesim Tasova, Hasan S.Z. Aksu

**Affiliations:** Cukurova University Faculty of Medicine, Adana, Turkey (F. Kuscu, A. Ulu, B. Kurtaran, S. Komur, A. Seza Inal, D. Erdogan, Y. Tasova, H.S.Z. Aksu);; Ankara University Faculty of Veterinary Medicine, Ankara, Turkey (O. Orkun)

**Keywords:** *Rickettsia sibirica mongolitimonae*, spotted fever group rickettsiae, ticks, Turkey, human, vector-borne infections, bacteria

## Abstract

In 2016, *Rickettsia sibirica mongolitimonae* was diagnosed for a man in Turkey. He had been bitten by a *Hyalomma marginatum* tick, from which PCR detected rickettsial DNA. Sequence analysis of the DNA identified *R. sibirica mongolitimonae*. Immunofluorescence assay of patient serum indicated *R.* *conorii*, which cross-reacts. PCR is recommended for rickettsiosis diagnoses.

The first case of human infection with *Rickettsia sibirica mongolitimonae* was reported in France in 1996 (*1*). The infection is called lymphangitis-associated rickettsiosis because of the lymphadenopathy and lymphangitis that occur with this infection but not with other spotted fever group rickettsioses (*2*). We describe a case of *R. sibirica mongolitimonae* infection with no lymphadenopathy and lymphangitis.

On May 1, 2016, a 53-year-old man was admitted to an emergency department in Adana, Turkey, for fever, headache, and maculopapular rash. He reported that 1 week earlier he had removed a tick from his umbilicus while farming in Adana, in the Mediterranean region of Turkey. He stored the tick in a glass jar and 2 days later sought care for high fever from his family doctor; administration of cefdinir produced no improvement. Four days later, he was hospitalized for fever (39°C), nausea, and malaise. Physical examination detected maculopapular rash and a black necrotic eschar at the center of an erythematous lesion on the patient’s umbilicus (Technical Appendix Figure, panel A). The patient had no sign of lymphadenomegaly or lymphangitis. Initial laboratory examination of serum showed 10.1 × 10^9^ leukocytes/L, 221 × 10^9^ thrombocytes/L, 13 g/dL hemoglobin, and 3.74 mg/dL C-reactive protein (reference range <0.5 mg/dL). A blood sample was sent to the National Microbiology Reference Laboratory in Ankara, Turkey. Doxycycline (100 mg 2×/d) was administered for suspected rickettsial disease. After 48 hours, the patient’s fever resolved, and his condition rapidly improved. He was discharged on day 5 of hospitalization, and doxycycline was stopped on day 10 after initiation.

Immunofluorescence assay of serum for typhus group rickettsiae IgM and IgG produced negative results. At the time of hospital admission, *R. conorii* IgM and IgG titers were 1:48 and 1:320, respectively. At a 1-month follow-up visit to the outpatient clinic, the patient’s *R. conorii* IgM and IgG titers had increased to 1:384 and 1:640, respectively.

The removed tick, provided by the patient, was stored in 70% ethanol and sent to the Protozoology and Entomology Laboratory of Ankara University Faculty of Veterinary Medicine for identification of the tick species and PCR (Technical Appendix Figure, panel B). Use of the morphological keys of Apanaskevich and Horak (*3*) led to tick identification as a *Hyalomma marginatum* female. DNA was extracted from the whole tick as described by Orkun et al. (*4*). Rickettsial DNA was detected by PCR with primers Rr. 190.70 and Rr. 190.701, which amplify the outer membrane protein A gene (*ompA*) of *Rickettsia* spp. (*5*). PCR and sequencing were conducted as described by Orkun et al. (*4*). The obtained nucleotide sequence was compared with sequences in the GenBank database, obtained by nucleotide sequence homology searches performed by BLAST analysis (http://www.ncbi.nlmn.nih.gov/BLAST). The gene sequence obtained in this study has been deposited in GenBank (accession no. KY513920).

PCR detected rickettsial DNA in the tick removed from the patient, and after sequence analysis, we determined that the rickettsial DNA belonged to *R. sibirica mongolitimonae*. According to nucleotide BLAST analysis, the obtained isolate is 100% similar to the reference strain *R. sibirica* subsp. *mongolitimonae* HA-91 (GenBank accession no. U43796) and *R. sibirica* subsp. *mongolitimonae* Bpy1 (GenBank accession no. KT345980) obtained from a biopsy sample from a human patient in Spain.

Although the climate and geography of cities like Adana in the Mediterranean region of Turkey are suitable for agents of Mediterranean spotted fever, we are unaware of any confirmed cases of *R. conorii* infection in this region. One reason may be limited access to diagnostic tools for rickettsial diseases. Another may be that doxycycline, the most effective treatment option for all rickettsial diseases (*6*), is easily administered for suspected cases of rickettsiosis with no differential diagnosis.

In Europe, *R. sibirica mongolitimonae* was detected in *Hyalomma excavatum* ticks in Greece and Cyprus; in *Rhipicephalus pusillus* ticks in France, Portugal, and Spain; and in *Rhipicephalus bursa* ticks in Spain (*6*). In 2016, *R. sibirica mongolitimonae* was isolated from 2 *H. marginatum* ticks in the central Anatolian region of Turkey (*7*). 

Nearly 35% of patients with *R. sibirica mongolitimonae* infection experience rope-like lymphangitis and other highly specific manifestations (*8*). The eschar on the patient reported here was located below the umbilicus, and he had no sign of inguinal lymphadenopathy or lymphangitis on the abdominal wall.

The best sample to use for detection of spotted fever group rickettsiae is skin biopsied from the inoculation eschar (*9*). We did not perform a biopsy because we had the vector tick removed from the eschar. Also helpful for rickettsiosis investigations are serologic analyses by immunofluorescence assay. In our laboratory, only *R. conorii* serologic tests are performed for spotted fever group rickettsiae; for the patient reported here, these test results were positive for *R. conorii*. However, cross-reactions are common among *Rickettsia* spp. in the spotted fever and typhus groups (*10*), and cross-reactions on serologic tests should be considered. Whenever possible, PCRs should be performed for rickettsiosis diagnoses.

Technical Appendix Eschar on the umbilicus of a man with *Rickettsia sibirica mongolitimonae* infection, Turkey, 2016, and *Hyalomma marginatum* female tick removed from the same man.
