# Supplementary material for: Rickettsia sibirica mongolitimonae Infection, Turkey, 2016
Source: Emerg Infect Dis. 2017 Jul;23(7):1214–6. doi: 10.3201/eid2307.170188 (PMC5512508; doi:10.3201/eid2307.170188)
Supplement: Technical Appendix — Eschar on the umbilicus of a man with Rickettsia sibirica mongolitimonae infection, Turkey, 2016, and Hyalomma marginatum female tick removed from the same man. [file 17-0188-Techapp-s1.pdf]

Article DOI: https://dx.doi.org/10.3201/eid2307.170188

# *Rickettsia sibirica mongolitimonae* Infection, Turkey, 2016

## Technical Appendix

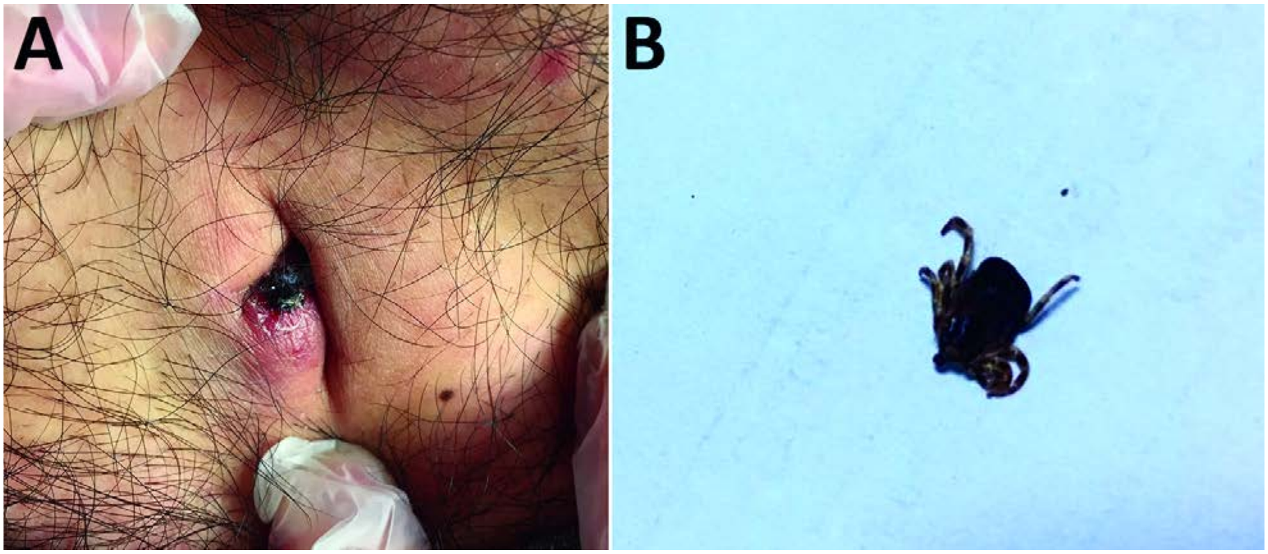


**Technical Appendix Figure.** *Rickettsia sibirica mongolitimonae* infection in man, Turkey, 2016. A) Black necrotic eschar on the umbilicus of the patient. B) *Hyalomma marginatum* female tick removed from the patient.
